# Lived experiences and coping mechanisms of parents following stillbirth and immediate postnatal death: an interpretative phenomenological study from a North Indian setting

**DOI:** 10.3389/fpsyt.2026.1736722

**Published:** 2026-03-02

**Authors:** Sonia Maurya, Krishna Kant Yadav, Pranay Vats, Krati Dixit, Moammar Hashmi, Shruti Bisht, Reema Mukherjee, Sarmila Mazumder, Barsha Gadapani Pathak

**Affiliations:** 1 Society for Applied Studies, New Delhi, Delhi, India; 2 Reproductive, Child Health & Nutrition (RCN), Indian Council of Medical Research, New Delhi, India; 3 Centre for Intervention Science in Maternal and Child Health, Centre for International Health, Department of Global Public Health and Primary Care, University of Bergen, Bergen, Norway

**Keywords:** bereavement care, immediate postnatal death, India, LMICs, meaning-making, parental coping, perinatal loss, stillbirth

## Abstract

**Background:**

Perinatal bereavement represents a profound rupture in parental expectations, often leaving families in an extended search for closure. While there is substantial research that highlight grief following perinatal loss, understanding how parents cope with such loss is essential for developing sensitive and contextually relevant bereavement care.

**Methods:**

This qualitative study explored the lived experiences of parents who had gone through perinatal loss within six months prior to data collection. Conducted during the formative and early implementation phase (Phase I and II) of the ongoing Strategies to Help in Optimal Pregnancy Outcomes and Reduce Stillbirths in India (SHRiSTI) project, between January and August 2025, in the Palwal district of North India. In-depth interviews (IDIs) were conducted with 15 bereaved parents, including both mothers and fathers. Using thematic analysis followed by Interpretive Phenomenological Analysis on selected narratives, the study examined how bereaved parents articulated and made sense of their loss, providing insights into their emotional, social, and cultural experiences of grief and coping. The COREQ checklist was used to ensure comprehensive reporting.

**Results:**

Four main themes emerged: parents’ responses, amplifiers of grief, coping mechanisms, and search for closure. Immediate reactions included fainting, shock, and planning legal action, while long-term responses involved sadness, loneliness, sleeplessness, and persistent thoughts of the child. Grief showed gendered patterns. Mothers expressed emotions openly, whereas fathers often internalized them. Delayed or inadequate care, unclear communication, and disrespectful treatment by providers, along with delayed disclosure, intrusive questioning, and stigmatization at family and community levels, further intensified grief. Coping strategies included memory-making, withholding memories; and digital remembrance emerged as a contemporary form of memorialization. Most parents struggled to find closure.

**Conclusion:**

Coping with perinatal loss is a negotiated and context-dependent process rather than a uniform trajectory toward closure. The study emphasizes the need for context specific and culturally sensitive bereavement care within maternal and newborn health services.

## 1 Introduction

All societies see death as a transition for the person who dies. How people prepare themselves for this transition and how the survivors feel and behave after a death has occurred varies a great deal. Crying, fear and anger are so common as to be virtually ubiquitous and most cultures provide social sanction for the expression of these emotions in the funeral rites and customs of mourning (1). The situation of a person who has recently experienced the loss of someone significant- notably a parent, partner, sibling, or child through that person’s death is referred as bereavement (2). Loss through death is by no means an experience restricted to the elderly. Pregnancy is a significant event in the life of an expecting mother, and perinatal loss leads to what is recognized as perinatal bereavement (PB). PB is the experience of parents that begins following perinatal loss which has been referred to as the loss of a fetus or baby due to a miscarriage, termination of pregnancy due to fetal anomalies, stillbirth, or neonatal death (3). It has been commonly defined as loss of an infant through death via unintended or involuntary loss of pregnancy by miscarriage, early loss (<20 weeks), stillbirth (≥20 weeks gestation) or neonatal loss (newborn through 28 days of life) (4). As of 2019, there were 2 million stillbirths per year, and a further 2.44 million deaths within the first month of life, 75% occurred in the first week of life and 1 million newborn died in the first 24 hours. Low- and middle-income countries (LMICs) particularly in sub–Saharan Africa and South Asia carry the highest percentage of global burden, 77% of stillbirth, and 81% of neonatal death (5). These figures only suggest the significant number of perinatal deaths that occur each year. Perinatal loss is generally regarded as the most painful form of bereavement because it is unexpected, often sudden and sometimes unexplained (6). It causes a range of painful emotional reactions, requiring grieving parents to respond to them. Short-term reactions include shock, anger, emptiness, helplessness, and loneliness; long-term reactions may include depression, anxiety, and post-traumatic stress disorder (PTSD) (7, 8).

All men and women who suffer perinatal loss essentially go through a PB process. Because of the intensity and implications of the loss experience, the large number of people it touches, and the systematic variations with which its consequences are distributed across populations, PB is a social issue of considerable importance (2). Out of the various kinds of pregnancy loss, stillbirth is one of the most challenging situations where a mother has to deal with the emotions of birth and death simultaneously. Globally, research on the lived experience of stillbirth, particularly in High-Income Countries and certain LMICs, highlights themes of profound grief, social isolation, and enduring psychological distress (9, 10). However, while these studies provide an insightful understanding of the lived experiences of bereaved parents following stillbirth, they are inadequate to capture the unique sociocultural and systemic nuances present in the Indian subcontinent. Despite India having the highest absolute number of stillbirths globally, the subjective emotional experiences of these parents remain largely overlooked in global research (11, 12). This study seeks to address this gap.

The data for this study has been collected as part of an ongoing Implementation Research (IR) to reduce stillbirth in India, which found that while state-led programs such as the Pradhan Mantri Surakshit Matritva Abhiyan (PMSMA) focus on reducing maternal and infant mortality, the focus on the bereavement care for affected families remain limited (13). With the launch of the Stillbirth Surveillance and Response initiative by Government of India in July 2025 which for the first time formally establishes the principles for bereavement care after stillbirth, attention to stillbirths has strengthened; however, current frameworks continue to prioritize prevention and reporting over psychosocial support for bereaved parents (14). In this context, there remains a critical lack of empirical evidence capturing how bereaved parents themselves experience, interpret, and navigate perinatal loss within these policy and programmatic environments. Understanding parental grief beyond biomedical and surveillance frameworks is essential for informing the development of responsive and culturally appropriate bereavement care.

This study makes a novel contribution to the literature on PB by examining the lived experiences of both mothers and fathers together, thereby addressing a critical gap in qualitative research on lived experiences of PB. While most prior work has explored maternal or paternal experiences separately, this study provides an integrated account of parental grief within the same social and cultural context, enabling comparative insight into shared and divergent experiential dimensions. In addition, by employing an interpretative phenomenological analysis (IPA), the research foregrounds the subjective, meaning-making processes of bereaved parents, revealing previously under-recognized emotional, relational, and cultural dimensions of perinatal loss. These insights advance understanding of culturally situated bereavement responses and highlight to the pressing need for culturally sensitive bereavement support interventions within maternal and child health policy in India (15, 16).

### 1.1 Definitional approaches to perinatal bereavement

Bereavement is a concern that extends beyond the boundaries of clinical interest; it affects at some point every family and raises logistical and policy issues for the health and social service agencies of every community (2). PB is characterized by a complex emotional response, most manifested as grief in both the mother and father, but often expressed differently between them, both in intensity and duration. The grief response of PB is influenced by situational, internal and external factors.

Often, PB is interchangeably used with terms like grief and mourning. Distinguishing between bereavement, grief and mourning, Stroebe and Hansson ( (2) emphasized that where bereavement is the objective situation of having lost someone significant; grief is referred to the emotional response to one’s loss. On the other hand, the actions and manner of expressing grief is known as mourning which often reflect the remembrance practices of one’s culture. Grief, which has been measured to know the bereavement, is a symptom of bereavement (2, 7). However, it is not the only response to bereavement (17). It is the emotional reaction that follows the loss of a valued other, or a normal response characterized by intense and deep sorrow that may be manifested in psychological, physical, behavioral or social ways. Similarly, mourning has been noted as a time-limited and dynamic expression of bereavement, varying from person to person and across time. It is further described as the manifestation of culturally patterned behaviors during bereavement that incorporate the experience of the loss into the outward expression as life is lived (18).

PB as a concept emerged in the 1970s; until then, the death of a baby, whether by miscarriage, stillbirth, or neonatal death, was not recognized by the medical community as a meaningful loss (19). With growing demand for reproductive rights, advances in abortion-related medicine, and increased clinical deliveries, reporting of fetal, perinatal, and neonatal deaths rose globally. Consequently, perinatal loss and its associated bereavement became a focus of interdisciplinary study.

The seminal work of Kennell et al. (19) provided the first empirical evidence that women experience significant mourning following infant death. This recognition spurred extensive research on PB through the 1980s (18, 19). Since this period, several self-report tools were designed to measure grief symptoms (2, 19–25). The development of these measurement scales was grounded in broader theoretical frameworks that sought to explain how individuals experience and cope with loss. Stroebe and Hansson ( (2) outlined three key theoretical approaches to bereavement: psychoanalytic, attachment, and stress theories. Drawing on Freud (26), the psychoanalytic view sees grief as a cognitive process of “grief work,” where the bereaved gradually detach emotional energy from the deceased (25, 26). Bowlby’s (1969) attachment theory interprets grief as a biological response aimed at restoring closeness to the lost attachment figure, while cognitive stress theory frames bereavement as a stressful life event whose impact on health can be moderated by coping resources such as social support (2, 27). While these scales have informed understandings of loss, less attention has been given to how parents experience and make sense of perinatal bereavement within their socio-cultural contexts. This study addresses this gap through an interpretative phenomenological examination of mothers’ and fathers’ lived experiences.

## 2 Methods

### 2.1 Study design and setting

This study employed a dual-phase qualitative analysis design to understand the lived experiences of parents who had experienced either stillbirth or the death of newborn within 24 hours of birth. The Phase I utilized Braun & Clarke’s six-step method to identify broad patterns, while Phase II (IPA) explored the idiosyncratic, lived experience of loss. The IPA was chosen because it enables in-depth exploration of how individuals experience, interpret, and give meaning to their loss (28). The final interpretation was guided by an integrated theoretical framework: the Dual Process Model (DPM) was used to categorize themes into ‘Loss-Oriented’ and ‘Restoration-Oriented’ stressors, while Continuing Bond Theory provided the lens for understanding how participants maintained connections with their infants (29). The study followed the consolidated criteria for reporting qualitative research (COREQ) checklist (30) to systematically enhance clarity, transparency, and rigor in qualitative research reporting, which has been provided as **Supplementary Table 3**.

The study was conducted in Palwal district of Haryana, a state in north India, as part of the SHRiSTI Project, an initiative supported by the Indian Council of Medical Research (ICMR). The broader SHRiSTI project is a multi-site IR program operating across six states of India (Karnataka, Maharashtra, Rajasthan, Meghalaya, Uttar Pradesh, and Haryana) to generate context-specific evidence for stillbirth prevention (31). The present study, however, aims to deepen understanding of how parents experiencing perinatal loss make sense of their bereavement and cope with loss within a specific socio-cultural context.”

The objective of this study is to examine parents’ lived experiences and responses following perinatal loss, and to explore the factors shaping their grief and coping strategies. In the present study, PB refers to the loss experienced by parents due to perinatal death, encompassing both stillbirths and early END. For this research, perinatal loss is operationally defined as the death of a baby either before or within 24 hours after birth. Stillbirth is defined as intrauterine death (IUD) occurring at or beyond 28 weeks of gestation, where the fetus shows no signs of life at birth. END, in this context, refers specifically to the death of a live-born infant within the first 24 hours of life. This definition is adopted to maintain consistency in case selection and to focus on the most acute period of perinatal loss that has significant emotional and psychological impact on the parents.

The study participants were identified through a multi-step process. First, the details (name of the parent, date of delivery, contact number) of cases with stillbirth and END were extracted from the delivery registers at the District Hospital (DH), Community Health Centers (CHCs), and Primary Health Centers (PHCs). Along with this, the cases of Home delivery or private delivery were collected from the Accredited Social Health Activist (ASHA) workers by two research team members. A combined linelist which included detailed list of cases of stillbirth and END was prepared from the collected information by the research team member with a degree of B.Sc. Nursing. Later, this list were handed over to the project coordinators with master’s in public health, who compiled the lists and were responsible for confirmation of the cases for reported pregnancy outcomes via telephonic interviews with bereaved parents (mostly fathers they received the calls in most cases. Afterwards, another research team member with B.Sc. Nursing was responsible for conducting a Verbal Autopsy and Social Autopsy (VASA) interview with all the mothers with stillbirth and END cases using the World Health Organization (WHO)-VASA Tool which confirmed the case’s stillbirth or END status (34). The final list was then prepared by the project coordinator and followed via telephonic interview by three other team members with graduate degree. The identified and confirmed cases with different outcomes of perinatal loss were contacted one week before the day when the in-depth interviews (IDIs) were conducted.

### 2.2 Sampling strategy and study participants

Given the idiographic orientation of phenomenological analysis, purposive and relatively homogenous sampling was prioritized. Homogeneity in qualitative research does not imply uniformity but rather ensures that participants share key characteristics related to the phenomenon of interest, which facilitates in-depth exploration and comparison across cases (32, 33). Inclusion criteria were bereaved parents (both mothers and fathers) aged 18 years or older who had experienced a stillbirth or END within the preceding six months from the date of interview. Participants were required to be permanent residents of the study area, able to provide informed consent, and available for an IDI at the time of data collection. Exclusion criteria included parents who had permanently relocated outside Palwal district, could not be contacted after repeated attempts, or declined to participate in the study. The restriction by timeframe of loss, geographic location, and socio-economic context ensured relative homogeneity of the study population.

### 2.3 Data collection

**Figure 1:**
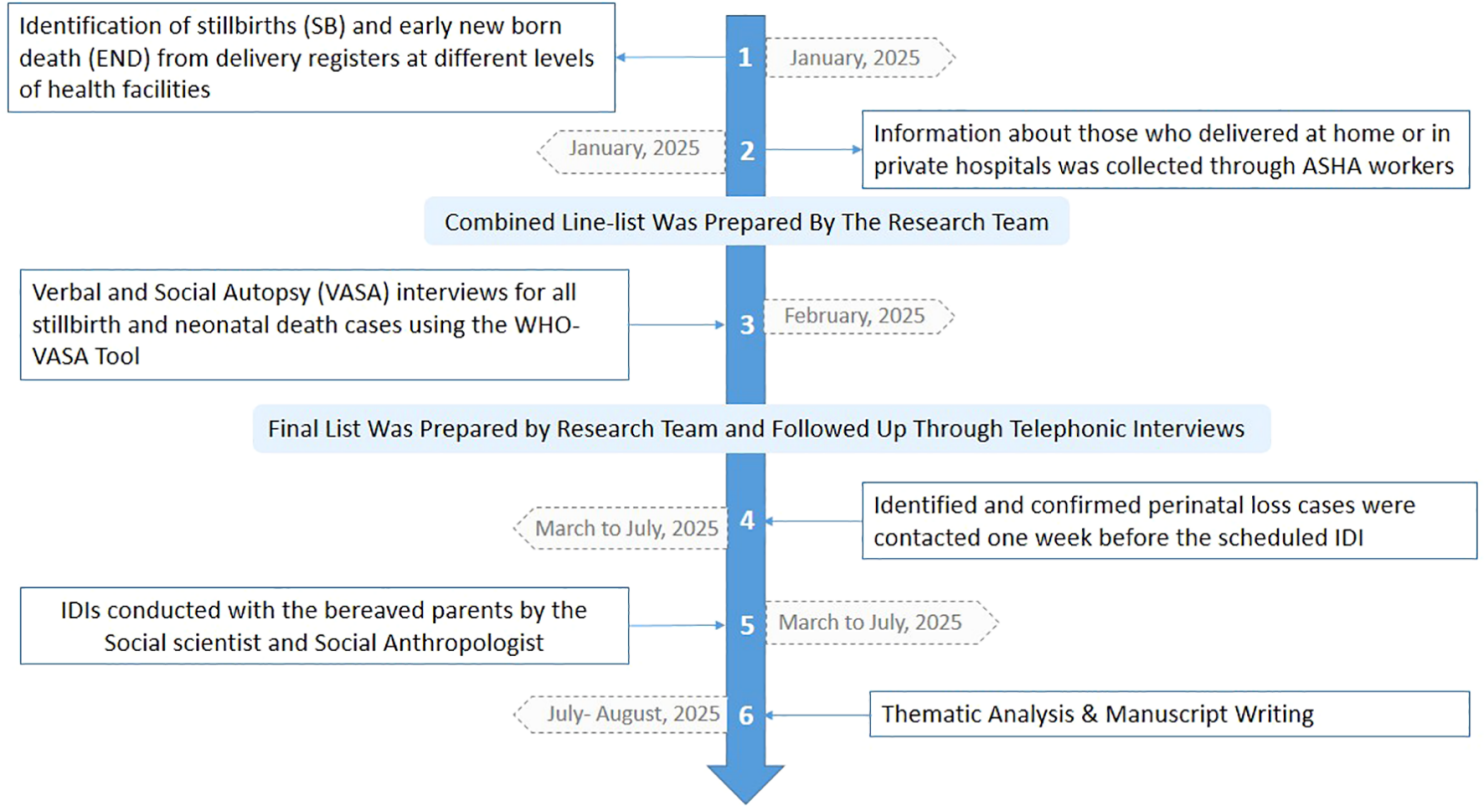
Process and timeline of data collection.

IDIs were conducted between January and August 2025 with bereaved mothers and fathers. To ensure cultural appropriateness and participant comfort, mothers were interviewed by a female social scientist and fathers by a male qualitative researcher, both holding and pursuing PhDs in Anthropology respectively. A male and a female team member with graduate degree accompanied the interviewers. They were responsible for note-taking, documenting the interview context, and recording non-verbal cues, which were later used to supplement the transcripts. Each interview was scheduled in advance at a time convenient for the participant and was conducted at their home, workplace, or another private location of their choice. In the case of working mothers and fathers, interviews were often arranged early in the morning before 10:00 AM or on Sundays to accommodate their work schedules. A total of 30 IDIs (15 mothers and 15 fathers) were conducted across different blocks of the district. Each interview lasted between 45 and 75 minutes, was audio-recorded with the participant’s consent, and supplemented with detailed field notes. The process of data collection continued until ideographic completeness was achieved, defined as the point at which no new themes emerged from the narratives. The timeline of data collection has been provided as **Figure 1**.

### 2.4 Data collection tool

Separate IDIs were conducted with bereaved mothers and fathers using pre-structured interview guides developed individually for each group. The guides were prepared after reviewing existing literature, international guidelines, and national documents on maternal health and bereavement support, and through consultations with subject experts in community medicine, obstetrics and gynecology. Additional inputs were incorporated from experiences narrated during social autopsy and positive deviance interviews undertaken in the larger project (35). The guides explored the bereavement experience of the parents, their pregnancy journey, the circumstances surrounding the loss, interactions with the health system, communication by healthcare providers, and the emotional reactions and support received from family and health workers. They also covered the struggles and challenges faced by the participants in physical, psychological, social, cultural, and financial domains, the coping mechanisms adopted individually and collectively within the family and community, the factors that amplified their grief, and their perceptions regarding the availability, gaps, and need for bereavement care and support.

Along with the qualitative guides, a short socio-demographic tool with pre-structured closed-ended questions was administered before each interview. This tool captured background information, including the age and education of both parents, family structure, monthly income, adverse pregnancy history, previous stillbirths, gender of the child, gestational age at the time of loss, mode of delivery, and place of delivery. The interview guides were pilot tested with a two bereaved parents outside the study area to ensure clarity, sensitivity, and cultural appropriateness. Feedback from these pilot interviews were used solely to refine the phrasing, sequencing, and framing of questions, resulting in the final version of the interview guides, which are provided in **Supplementary Annexures 1**, **2**.

The researchers were aware that their own positions could influence both the process of interviews and the interpretation of narratives. The two primary interviewers were trained anthropologists with doctoral-level expertise, one specializing in maternal and child health and the other in ethnographic research. The male researcher’s ethnographic training focused interviews on cultural practices and contextual meaning, while the female researcher’s maternal mental health expertise guided exploration of emotional experiences and psychological impacts. While they did not share the personal experience of perinatal loss, they were sensitized and well equipped to issues of grief, gender, and family dynamics through their disciplinary training. Both interviewers were relative outsiders to the local community, which helped participants speak without fear of local judgment, but it also required building rapport and trust at the start of each interaction. Research team members, drawn from similar cultural backgrounds as the participants, further bridged this gap by providing contextual understanding and helping the team remain grounded in the lived realities of the community. To promote comfort and openness, the interviews were conducted in locations chosen by participants, most commonly their homes, although in some cases, particularly for fathers, interviews were conducted at their workplaces. Privacy was prioritized by requesting family members not to be present during the interviews, and flexible arrangements, such as rescheduling or shifting to quieter locations, were made when privacy could not be ensured.

Given the sensitive nature of pregnancy loss, a previously developed response guide was used by the interviewers for addressing emotional distress during the IDIs (34). This document titled as Solace Protocol, designed to guide researchers on how to respond/react during data collection for distress management, was carefully followed, which has been provided in **Supplementary Annexure 3**. If a participant showed signs of emotional distress during the discussion, the interviewer paused the interview, provided reassurance, and resumed only with the participant’s consent. To maintain rigor and reflexivity, the research team held debriefing meetings every two days to review field notes, discuss emerging themes, and refine the interview guide as needed. Aware that our backgrounds could shape how we engaged with bereaved parents and interpreted their experiences, interviewers maintained personal notes after each interaction, reflecting on emotions, assumptions, and challenges. These reflections were discussed in team meetings to remain self-aware and minimize bias. While the interview guide offered structure, conversations flowed naturally, allowing participants to speak freely and at their own pace. Non-verbal cues and contextual details were carefully noted to preserve meaning of the narratives. By combining careful listening, respectful probing, and ongoing team discussions, we sought to ensure that the accounts presented in this study remained close to what parents wanted to share.

### 2.5 Data analysis

All interviews were audio-recorded with participants’ consent, transcribed verbatim in the Hindi language, and subsequently translated into English by the same researchers who conducted the IDIs. This process ensured fidelity of meaning and preservation of cultural nuances. The interview guide was organized around the study’s objectives, with broad sections addressing responses to loss, coping strategies, and support needs. However, interviews were conducted in a flexible and participant-led manner, allowing respondents to elaborate freely on issues they considered important, and enabling the conversation to move beyond the pre-defined themes as new insights emerged. This ensured coherence between the thematic and IPA. We conducted this dual analysis of data set by applying thematic analysis to transcripts from the full sample and IPA to sub-sample for identifying the individual perspectives. This combined approach allowed both an exploration of emergent themes and an in-depth understanding of participants’ lived experiences.

In the first stage, a deductive approach was followed for conducting thematic analysis using Braun and Clarke’s six-step method (36). All the transcripts were first uploaded into NVivo software version 15. In the first step, the two qualitative researchers (SM and KKY) read the transcripts independently to become familiar with the data. In the second step, they generated initial codes by tagging meaningful segments of text related to experiences of pregnancy, immediate and extended responses to the perinatal loss, coping mechanism, and perceptions on the need for bereavement care and support. This iterative process was followed by a joint review in the third step, where codes were grouped into potential themes and sub-themes, using NVivo’s coding dashboard. In the fourth step, generated themes and sub-themes were refined and cross-checked against coded verbatim extracts. In the fifth step, these themes were clearly defined with operational descriptions, and finally, in the sixth step, the results were tabulated with supporting verbatim and interpretations. However, during this process some cases showed the in-depth, pragmatic and existential experiences. These cases were subsequently re-examined using an IPA approach. This approach helped in engaging deeply with the idiographic accounts of parents. The process involved reading and re-reading individuals’ verbatim, developing exploratory comments, identifying emergent themes within each case, and then examining patterns of convergence and divergence across cases. This idiographic focus allowed the research team to remain grounded in the unique experiences of each parent, while also drawing out shared meanings among the bereaved parents. The IPA was followed using the Dual Process Model of Coping with Bereavement and the Continuing Bonds theory (37). This helped to interpret emergent themes in relation to existing theoretical understandings. This process generated two new themes which were ‘Amplifiers of grief’ and ‘need for closure.’ Cases were selected for IPA based on narrative richness and depth rather than representativeness, consistent with the idiographic commitment of phenomenological inquiry. The IPA was not used to validate themes generated through thematic analysis, but to deepen understanding of meaning-making processes.

**Figure 2:**
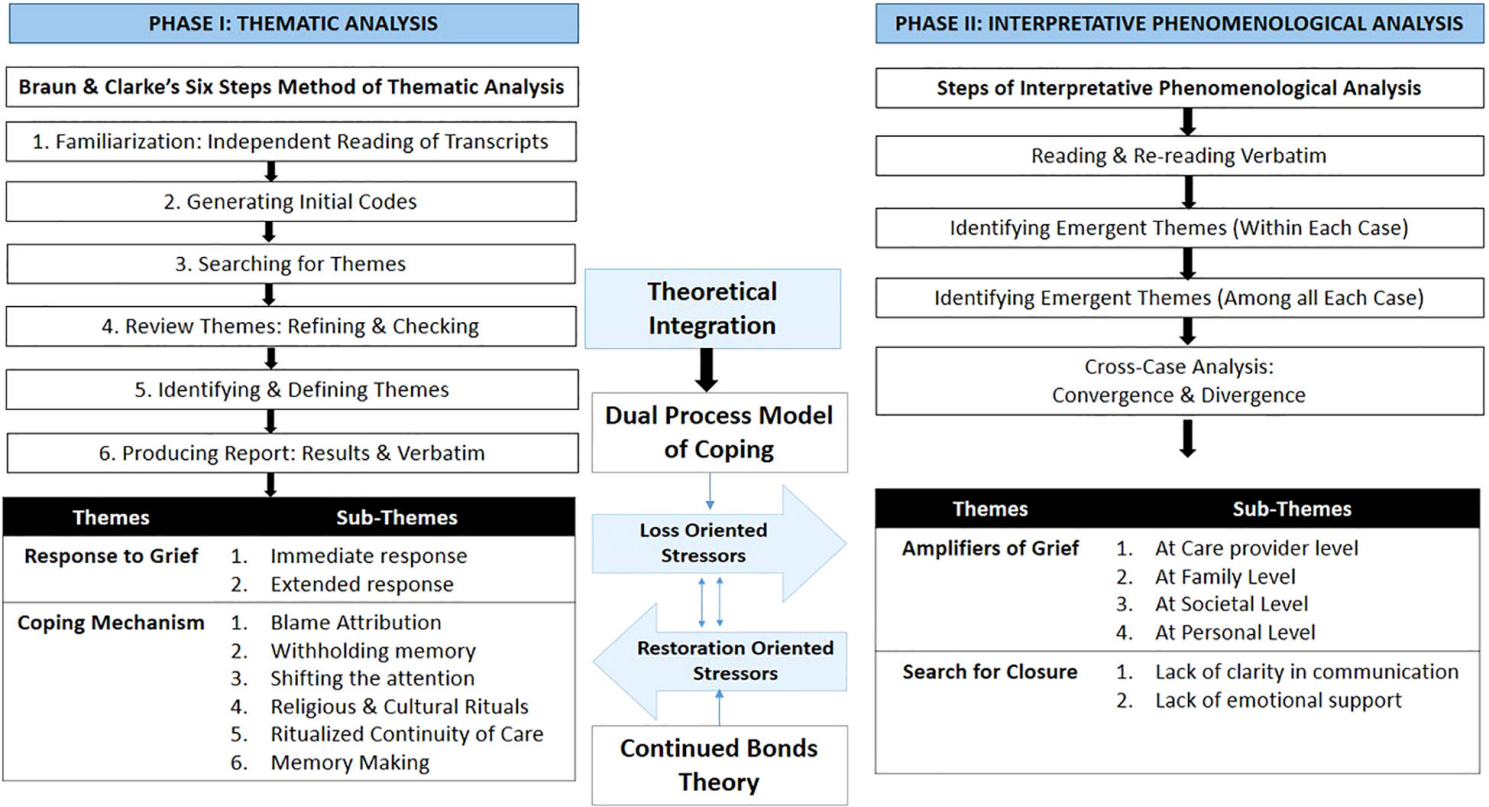
Process of qualitative data analysis.

By combining thematic analysis and IPA, the study sought to balance breadth with depth: capturing common patterns while preserving the individuality and emotional richness of bereaved parents’ experiences. The findings were organized around two broad types of stressors, as conceptualized in the Dual Process Model of bereavement: loss-oriented and restoration-oriented stressors. Loss-oriented stressors referred to experiences that drew parents’ attention directly to perinatal loss and its emotional and traumatic consequences. Restoration-oriented stressors, in contrast, reflected parents’ efforts to adapt to life after the loss, including attempts to cope, reorient daily life, and make sense of the absence. Within this analytic framing, four interrelated themes were identified. Loss-oriented stressors were captured through the themes of *Response to Grief* and *Amplifiers of Grief*, while restoration-oriented stressors were reflected in the themes of *Coping Mechanisms* and *Search for Closure* (38). The complete process of data analysis has been provided in **Figure 2**.

## 3 Results

### 3.1 Socio-demographic and health characteristics of study population

The socio-demographic and health characteristics of the participants provide essential context for understanding the experiences of families who recently faced stillbirth or END. Most mothers were young, with 60% falling in the 18–25 years age group (mean age: 25.74 ± 4.58 years), while the mean age of fathers was 30.86 years [Standard Deviation (SD) ± 6.36], with most (73.3%) between 26–35 years. In contrast to mothers (33.33%), the majority of fathers (73.33%) were literate, underscoring a gender gap in educational opportunities. Most mother were homemakers and lived in joint family households (80%). Nearly half of the families reported a monthly income of ≤10,000 INR, indicating low socio-economic status. Regarding obstetric history, 60% of mother were multigravida and 20% were primiparous. One-third (33%) of babies were delivered before 37 weeks of gestation. The majority of deliveries (80%) occurred through normal vaginal delivery, and 60% of births took place in private hospitals (**Table 1**).

**Table 1:** Socio-demographic and health characteristics of study population.

Name of the characteristics	N= 30 participants [15 parents]	Mean (± SD)
	Count (%)	
Age of mothers (years)		
18-25	9 (60)	25.74 (4.58)
25- 35	6 (40)	
Age of fathers (years)		
18-25	4 (26.67)	30.86 (6.36)
25- 35	11 (73.33)	
Education of mothers		
Literate	5 (33.33)	
Illiterate	10 (66.67)	
Education of fathers		
Literate	11 (73.33)	
Illiterate	4 (26.67)	
Family structure		
Joint	12 (80)	
Nuclear	3 (20)	
Family income		
≤10,000	7 (46.67)	
10,001-20,000	7 (46.67)	
20,001- 30,000	1 (6.67)	
Pregnancy and health-related variables		
Gravida		
Primiparous	3 (20)	
2^nd^ & 3^rd^ pregnancy	3 (20)	
≥4 pregnancies	9 (60)	
Gestational age		
Pre-term birth (≤37 weeks)	5 (33.33)	
Full-term birth (37-41 weeks)	7 (46.67)	
Post-term birth (>41 weeks)	3 (20)	
Mode of delivery		
Normal	12 (80)	
C-section	3 (20)	
Place of delivery		
Home	1 (6.67)	
Public Hospital	2 (13.33)	
Private Hospital	9 (60)	
Others (Midwives & Local doctors)	3 (20)	
Gender of baby		
Female	8 (53.33)	
Male	7 (46.67)	

All the women were Homemakers.

### 3.2 Qualitative findings

The thematic analysis of all interviews followed by a more detailed IPA of selected in-depth interviews, highlighted how multiple social, relational, and experiential factors come together to shape the bereavement process within the study community. Four major themes emerged from our thematic and IPA analysis which included parents’ response following their loss and coping mechanism (**Table 2**) and amplifiers of grief, and the search for closure (**Table 3**) respectively. Parents’ response to their loss meant their immediate and long term emotional, psychological and physical reactions after the loss of their child. Amplifiers of grief meant the internal and external factors that intensified the experience of grief and prolonged the process of healing. Coping mechanism was the strategies parents used to manage their grief, including emotional expression, seeking support, and finding meaning in their loss. The last theme which was search for closure refers to the parents’ ongoing emotional struggle to find understanding, resolution, and acceptance after their loss. The theme wise narratives have been given in the **Supplementary Table 1**. Following sections discuss these themes in detail.

**Table 2:** Summary of thematic analysis results.

Theme	Sub-theme	Codes	Mothers (N=15)	Fathers (N=15)
			Count (%)	Count (%)
Response	Immediate	Faint	4 (26.7)	1 (6.7)
		Shock	6 (40.0)	5 (33.3)
		Cry	10 (66.7)	2 (13.3)
		Shock and dissonance	5 (33.3)	4 (26.7)
		Planning Legal Action	1 (6.7)	3 (20.0)
	Extended or long term	Sadness	13 (86.7)	10 (66.7)
		Headache	5 (33.3)	2 (13.3)
		Anxiousness	8 (53.3)	5 (33.3)
		Irritation and anger	6 (40.0)	8 (53.3)
		Loneliness	9 (60.0)	4 (26.7)
		Desire for escape and solitude	7 (46.7)	3 (20.0)
		Sleeplessness	10 (66.7)	6 (40.0)
		Persistent thought of the child	11 (73.3)	8 (53.3)
		Gendered expression of grief	8 (53.3)	9 (60.0)
		Gendered Attribution of Survival	3 (20.0)	5 (33.3)
Coping mechanisms	Religious/Cultural Rituals of mourning	Hawa and Mahak: Purity and pollution	8 (53.3)	3 (20.0)
		Idea of Din and Jannat: Theological framing of loss	6 (40.0)	6 (40.0)
		God’s own child: Faith-Based Meaning-Making	9 (60.0)	7 (46.7)
		Collective recitation of Sahada (declaration of faith)	5 (33.3)	4 (26.7)
		Visit to the grave	6 (40.0)	5 (33.3)
	Ritualised Continuity of Care	Offering breast milk at the grave: Embodied Rituals of Grief	3 (20.0)	0 (0.0)
	Memory making	Digital Remembrance	4 (26.7)	3 (20.0)
		Materializing the Baby’s Presence	6 (40.0)	5 (33.3)
	Withholding Memory	Erasure of material Memory	5 (33.3)	4 (26.7)
		Silencing of grief	8 (53.3)	6 (40.0)
		Emotional regulation	9 (60.0)	9 (60.0)
		Keeping Grief Inside	10 (66.7)	8 (53.3)
	Blame Attribution	Complain about care providers	6 (40.0)	5 (33.3)
		Systematic delay in Referral	4 (26.7)	3 (20.0)
		Grievance over health infrastructure and facility	5 (33.3)	6 (40.0)
	Shifting the attention	Self-talk & Negotiations with God	8 (53.3)	9 (60.0)
		Increased focus and attention on other child	7 (46.7)	6 (40.0)
		Returning to work	5 (33.3)	9 (60.0)

**Table 3:** Summary of interpretative phenomenological analysis.

Theme	Sub-theme	Codes	Mothers (N=15)	Fathers (N=15)
			Count (%)	Count (%)
Amplifiers of Grief	At Care Provider’s level	Caregiver’s avoidance	5 (33.3)	4 (26.7)
		Unresolved suspicions	3 (20.0)	3 (20.0)
		Delayed care leading to death	7 (46.7)	6 (40.0)
		Uncertainty about the time of death	6 (40.0)	5 (33.3)
		Place of delivery and decision making	4 (26.7)	3 (20.0)
		Experiences of Disrespectful and Neglectful Care	8 (53.3)	5 (33.3)
	At Family level	Delayed Disclosure	6 (40.0)	4 (26.7)
		Intrusive Care	5 (33.3)	2 (13.3)
		Domestic Violence	3 (20.0)	0 (0.0)
		Minimal, inadequate support	9 (60.0)	5 (33.3)
	At Societal level	Intrusive Social Enquiries	8 (53.3)	7 (46.7)
		Repeated Narration of the event	7 (46.7)	5 (33.3)
		Recurrent Conversations of Loss	6 (40.0)	5 (33.3)
		Avoidance and Stigmatization	5 (33.3)	3 (20.0)
	At personal Level	Daily Encounters with Loss	9 (60.0)	8 (53.3)
Need for closure	Lack of Clarity in Communication	Desire for clear guidance	10 (66.7)	8 (53.3)
		Conflicting explanations to the loss	9 (60.0)	7 (46.7)
		Lack of honest communication	8 (53.3)	6 (40.0)
		Absence of explanation	6 (40.0)	4 (26.7)
	Lack of Emotional Support	Counselling to the husband and family	9 (60.0)	5 (33.3)
		Need for Emotional Reassurance from Providers	10 (66.7)	8 (53.3)

#### 3.2.1 Immediate and extended response

Parents reported two kinds of response to their loss- immediate and long term response. Immediate response referred to the moment when learnt about the loss. Mothers experienced severe grief immediately after the death of their baby. Both mother and fathers described an initial phase of physical and emotional response which included getting faint, shock, crying, and dissonance when they first learnt about their loss. Some parents, especially fathers, responded with thoughts of legal action against the care provider which reflect their urgent need to make sense of the loss and process their anger and grief. The extended response which included the period between few days to up to six months in this case, bereaved mother reported the manifestation of grief through sadness, headaches, sleeplessness, persistent thoughts of the child, and a desire for solitude. Father’s commonly reported feelings of guilt and increased engagement in work following their loss. Emotional states varied between anxiety, irritation, anger, and loneliness, with grief often taking gendered forms. Father tended to attribute survival differently or silence grief, while mother expressed sadness and irritability more openly.

“Everyone is distressed. But mostly the mother suffers more. You also know men neither cry openly nor show it. That’s how it is. Most mother express grief. Men just sit quietly, looking sad.” [IDI-1; Father]

“Since I am the elder in the house and there is no one else above me, I had to understand it.” [IDI-2; Father]

The other responses highlighted how father in some cases actively tried to frame their emotional response as collective or shared grief. One participant stated,

“Look, anyone feels sorrow when a child dies. After living together, eating together, staying together throughout the pregnancy, of course, both mother and father feel the loss. The whole family feels it.” [IDI- 1; Mother]

However, in contrast to this, in some cases, fathers were equally devastated by the news of the loss and had similar responses to their grief. Notably, these were fathers who had been more actively involved throughout the pregnancy, regularly accompanying their wives for check-ups and ultrasounds.

“There is this PHC near our village. I used to go with her every month for check-ups. We also gave reports to the Anganwadi worker. It was never that we delayed it by a month or so, we went every month without fail. Because we didn’t even have one percent of doubt, we had gone so happily, like it was just another normal check-up. And then suddenly they told us there was this problem…. we couldn’t believe it. But when the second report came back around 10:30–11 am, the doctor said the baby was no more. You will have to get an abortion as soon as possible. I could not believe it. I felt like the ground fell out from beneath my feet. How could it happen? I was shattered. We both came out and started crying.” [IDI-3; Father]

Some bereaved mothers reported imagining alternative outcomes when discussing their loss by attributing the gender possibilities of survival. One mother said that if it was a girl child it could have survived.

“I keep thinking that it was such a good boy. If it had been a girl, she might have survived.” [IDI-3; Mother]

#### 3.2.2 Amplifiers of grief

During the analysis of some transcripts using IPA, parents described several experiences that intensified their grief. These were grouped into three categories: factors related to care providers, factors within the family, and factors at the societal level.

At the level of care providers, two bereaved mother reported that they were left in the same ward with other mothers with their newborn babies which increased their grief.

“I just couldn’t stay there … seeing them made me want to cry a lot … because I didn’t have my child … I didn’t tell anyone anything … No, I didn’t tell anyone; I only kept telling my husband and my sister-in-law again and again that take me away from here, I don’t feel comfortable here, everyone here has children, and I am like this here. Then my husband was saying we will leave for home only when the doctor gives leave.” [IDI-5; Mother]

A similar experience was shared by another mother who reported,

“After the delivery, all the mothers in the hospital were breastfeeding their babies. Seeing them gave me so much pain, I kept thinking, I would have been feeding my baby like that too. I was a mother who had just lost her child, surrounded by others holding theirs, breastfeeding them.” [IDI-8; Mother]

Others highlighted the unresolved suspicions, avoidance, delayed care, uncertainty about the time of death, and experiences of neglectful or disrespectful treatment as the primary reasons that amplified their grief.

“The nurse wrapped the baby in a swapy (baby sheet) and kept it on a tray. Then she told me that she (mother) needs to be kept under observation for 12 more hour. Please take the **“**body**”** from here. It was around 12 in the night. How could we take home right now? Unfortunately, that day, there was too much rain and thunder that night. I said where will we go right now? It is too heavily raining outside, can we take it when the rain stops? But the nurse was “not good”. She looked at the tray where the baby was kept and said “body cannot be kept here whole night.” I tried to explain her that our house was 10 km away, we would take it as soon as the rain stops in the early morning but she wasn’t ready to listen.” [IDI-3; Father]

Similarly another father who had very recently experienced his loss and was in much grief, said,

“We spent more than fifteen thousand in that hospital, but no one even sat with us to explain. This made me so angry and helpless at the same time.” [IDI-2; Father]

The father noted his discomfort with the nurse’s repeated use of the term *body*. Despite explaining the challenges of leaving during heavy rain at night, he was instructed to take the baby. While stillborn or deceased newborns are usually buried in his community, he had to cremate the baby the next morning, which, according to him, further intensified his grief.

Some parents retrospectively questioned their delivery-place decisions.

“I wasn’t there at the time. If I had been there, I would not have taken the baby to her (the nurse). I would have gone straight to the government hospital.” [IDI-1; Father]

At the family level, delayed disclosure emerged as a significant factor intensifying maternal grief. In the interviews, most mothers reported not being informed of their loss immediately. They described the postponement of disclosure, particularly by husbands and other family members as deeply painful, with several noting that such delays greatly magnified their suffering.

“Two days after coming from the hospital, I overheard my sister in law and mother in law talking with each other. That’s when I learnt about it. Since then, I kept crying I kept saying to myself that this was wrong, that they should have told me earlier. At that moment I felt so hurt; I kept thinking if they had told me sooner, maybe it wouldn’t have been so painful, maybe I could have handled it. They had hidden it, perhaps to spare me, but instead it felt like a betrayal. I didn’t even see or hold the baby once, not even once. Sometimes I think, if I had at least held it, I might have known what it would have been like; perhaps I would have cried differently, perhaps the images in my head wouldn’t keep coming back. Now I keep going over that day in my mind, again and again; some days it overwhelms me.” [IDI-2; Mother]

Thus, the secrecy and delayed disclosure not only deepened the mother’s grief, but also turned what was intended as protection into a sense of betrayal and exclusion. The absence of a final encounter or never seeing or holding the baby left the mother’s mourning unresolved with many counterfactuals of “what if”. The repeated mental reliving of that day illustrate how trauma is sustained through silence and denial of mother’s agency. Along with the delayed disclosure, some mother reported intrusive care within their families also amplified their grief by being repeatedly talked about the loss.

”I used to feel restless, as if I should go far away from here. Everyone was around me-my sister-in-law was there with me all the time, my husband’s aunt and my mother-in-law also stayed in my room. But still my mind didn’t settle with their conversations. They kept me engaged in their talk, yet I still felt like staying apart from them. They would often explain to me, saying, ‘Babita (changed name), don’t think so much. You will regret it later. Your eyes will hurt, your head will ache-your head is still fragile, your eyes are delicate. Don’t think so much.” [IDI-10; Mother]

Such quest for solitude or personal space was more frequently reported by bereaved mother who were living in a joint families where they felt they had little space to process their grief. Some bereaved mother also reported that they witnessed constant presence of someone with them to console them. Inadequate support and domestic violence were reported as other amplifiers in some cases. Two mother reported that the care and support they received during pregnancy was suddenly reduced following the pregnancy loss. This caused them much pain by intensifying their grief.

At the societal level, incessant enquiries and social interactions further compounded the mothers’ suffering. Many mother reported that after returning home, they were frequently visited by relatives and neighbors who persistently asked questions such as when and how the loss had occurred. The need to repeatedly narrate the circumstances of their loss became a recurring reminder of their bereavement, thereby intensifying their grief. Notably, this burden of social questioning was reported almost exclusively by mother, while fathers were rarely subjected to the same degree of scrutiny.

Some mother also reported experiences of being avoided by family members or neighbors, which they perceived as stigmatizing. In certain cases, they were excluded from social gatherings, particularly religious events, leading to feelings of isolation.

Such forms of social avoidance and stigma further deepened their grief and reinforced their sense of marginality. Notably, no fathers disclosed such experiences. In contrast, the triggers for them came from more personal experiences. One father shared his personal experience that the grave of his lost child which was in way to his work often came in his sight whenever he left his home, evoking grief and sorrow to him.

“I am unable to digest this. It just doesn’t go from my mind. Our burial ground is just on the way to my work, dragging my attention each time I pass through that route. In the initial few days, I unconsciously went to the grave just to check if the grave was safe. I was scared that the stray animals might dig it up or remove the soil from it. And in general I used to walk through that way for many days whenever this thought came to my mind.” [IDI-1; Father]

#### 3.2.3 Coping mechanisms

Coping mechanisms emerged as another important theme. Coping with their loss appeared as a profoundly complex process that unfolded within the personal, familial, and cultural contexts in which parents were situated. Distinct ethnic groups held their own cultural norms and traditional attitudes toward bereavement. Parents described their understanding and navigation of loss as influenced by shared cultural norms, values, and expectations, resulting in responses that were both personal and socially patterned. From the analysis, four broad forms of coping were identified: (1) religious and cultural rituals of mourning, and (2) ritualized continuity of care, (3) memory making, (4) withholding memory. These themes together resonated with Klass, Silverman, and Nickman’s (39) continuing bonds theory which challenges the notion that healthy grieving requires detachment from the deceased. Instead, it posits that bereaved individuals maintain an ongoing connection with the lost loved one through transformed and symbolic relationships. This perspective reorients grief support away from a “letting go” paradigm toward an understanding of bereavement as an adaptive process in which the relationship endures, albeit in altered forms, between the living and the deceased (39).

##### 3.2.3.1 Religious/cultural rituals of mourning

The first stage is of religious and cultural ritual of mourning which include the last rites of the dead and following services. Respondents from both ethnic groups (Hindu and Muslim) described to follow the practice of burial in cases of stillbirth or END followed by observing three to five days of religious rituals which included performing puja, *‘Hawan’* and *‘Yagya’* along with offering prayers or recitation of faith.

“For a small child, the rituals usually last only 3–4 days. For adults, it goes on longer up to 13 days. We had to perform the rites. We performed a ‘*Hawan*’ and sprinkled holy water from Ganga in the house, that’s all. All of this was done on fifth day. Until then, we were at home, didn’t go anywhere. During that time, food wasn’t cooked at home for a day or two, maybe 2 or 3 days. The ‘*Puja’* was probably done on the 5th day. After that, from the next day, we resumed our usual work.” [IDI-6; Mother]

When asked about the role of such rituals in coping, the bereaved father said,

“It didn’t feel like anything at all. It just felt like our daughter suddenly disappeared. People do *puja* and rituals for peace of mind, but it’s not like it helps you forget. That peace of mind doesn’t really come from anyone. When someone passes, whether a child or an adult, you still have to follow the rituals that are prescribed. Otherwise, people start talking: this wasn’t done, that wasn’t done. But the truth is, the pain doesn’t go away. Yes, maybe the grief for an adult is felt more deeply, and for a child, maybe a little less, but the sorrow remains either way.” [IDI-3; Mother]

Another participant shared, “We didn’t do anything at that time. We only performed a small ‘hawan’ after ‘sawa mahina’ (about five weeks) had passed.” When asked about the purpose of the ritual, he explained, “People say it helps to clear the ‘hawa’ (presence) and ‘mahak’ (lingering essence) of the deceased.”

##### 3.2.3.2 Ritualized continuity of care

In some cases, participants described a ritualized continuity of care carried out by the family and the community, involving embodied expressions of grief and associated practices. One mother recounted:

“After the baby passed away, we performed the ‘hawan’. My breast milk was then taken to the cremation ground where the child was buried. The priest had also advised us to do this during the ritual, reminding us that the soul of a baby should not suffer from hunger. So both at the time of burial, and again on the second and third days, my husband went to the cremation ground and placed the milk at the grave.” [IDI-6; Mother]

When asked whether she had ever visited the site herself, she responded:

“I have never gone there, not even once. In our culture, women are not allowed to go. It gave a little peace of mind, to feel that even in death, we were still feeding our child. My husband, though silent, supported every ritual, making sure it was done properly. For him too, it was a way of showing care, even though the baby was no longer with us.” [IDI-8; Mother]

A similar practice was narrated by another mother who reported that male in her family and community visit the grave for collective recitation of sahada (declaration of faith) while female do the same at home. The husband of the same mother said,

“In our deen (faith), it is believed that whatever happens is for the good. According to our beliefs, a child below the age of eight is considered to go straight to jannat (heaven), he is not held accountable like adults, who face either heaven or hell. So we are taught to accept it with patience. But still, it’s true that everyone longs to hold the child just once, to feel that embrace.” [IDI-8; Father]

In addition, some parents tried to distract and reorient themselves by focusing on other children or returning to work. One father reflected,

“I just thought I already had one son and one daughter, so I got the operation done. I said to my wife, we already have two children … what else is there to do now? Let’s get the operation done. But the doctor refused to do it now, otherwise I would have gotten it done. Now there are two children, and we have to think only about them.” [IDI-1; Father]

Some parents sought refuge in expressing their grief through attribution of blame, repeatedly complaining to health care providers and institutions for their loss. Others reported turning inward, engaging in ‘self-talk’, prayer or private reflection. A distinct pattern emerged among fathers, many of whom described shifting their focus toward supporting their partners. One father reflected:

“My bond with her (wife) is stronger now, more than before. Because when you see a person go through such a situation, like I saw her in the hospital, then only you truly understand them.” [IDI-3; Father]

Some parents who did not have other children or supportive partners described a deep sense of emptiness and a need to hold onto memories of the lost child.

##### 3.2.3.3 Memory making

Some parents reported frequently viewing photos when feeling low after their loss. Almost all bereaved mother reported to have picture of the lost child in their phone galleries and see these pictures whenever they missed their child. One mother said,

“What if she is not here? At least I have her memories with me. Whenever I feel low or miss her, I see her picture which I had posted on Instagram after she was born.” [IDI-13; Mother]

Another mother reported,

“We performed a ‘hawan’ at home after the baby passed away. Since I was still in my postpartum period and considered ‘unclean’, the photo of the baby was removed from where it was kept and placed in another room. I asked for the photo to be left with me. My husband understands this attachment. Even though the elders follow traditions of purity and rituals, he quietly supported my wish to keep the baby’s memory alive in our home.” [IDI-7; Mother]

While some parents sought solace through shared expressions of grief, others coped by deliberately distancing themselves from reminders of the loss.

##### 3.2.3.4 Withholding memory

Some parents described sharing their grief with others, while others chose to distance themselves from reminders of the loss. Some parents, mostly fathers, mentioned avoiding photographs, removing belongings, or not speaking about the event. One father reported that in his community, all material memory associated with the dead or either discarded or given up-

“All her clothes, medicines, tablets, anklets, whatever was there, we removed it all from our house. After performing the last rites, when I returned home, I tore up and threw away all the papers, reports, ultrasounds, everything. The clothes and the bed sheet were given to the bhangi (a term used for the sweeper). Whatever the child had used, we put it all out. This is what happens with the material memory of everyone in our culture, whether it is a child or an adult.” [IDI-1; Father]

Many mothers saved and later viewed photos on phones or social media (digital remembrance). However, the husband of one such woman revealed that he had deleted all the photos from the phone, explaining that seeing any reminders of the lost child caused him pain, as his wife would keep looking at them and crying.

“I deleted the photos that were on my phone, but the ones that had been put on Instagram at the time, those won’t get deleted so easily. Then, the photos I had taken to send to the nurse were also there. I removed all of them. I didn’t like seeing her crying all the time by seeing those pictures in the phone. “ [IDI-1; Father]

While some parents coped by distancing themselves from memories, others managed their grief by directing frustration and anger toward health care providers, institutions, or circumstances as a way to make sense of their loss.

##### 3.2.3.5 Blame attribution

Many bereaved parents, particularly fathers, voiced grievances against doctors, ASHAs, and the broader health system, citing negligence, delays in referrals, and inadequacies in infrastructure.

One parent complained that the doctor never checked the child’s heartbeat during any of the previous visits and only did so after the child had died. They felt that the child might have survived if the heartbeat had been monitored regularly by the doctor.

“We had been coming for regular check-ups so many times, but she never checked the heartbeat. It was only when the child died that they checked the heartbeat. Our child could have been saved if they had regularly checked the heartbeat during the previous visits.” [IDI-6; Mother]

One father who spoke very little throughout the interview and responded to most questions in silence, asked the interviewer if there is any way to complain against the ASHA worker. In a similar response, one mother described ASHA’s visit as mechanical, intrusive and apathetic.

“She came only after getting instructions from above. I was still in the bathroom when she arrived, and she didn’t even step inside the house. She simply glanced at the child, quickly jotted something in her register, and left. While I was bathing, she even forced the gate open and didn’t allow me to finish properly. It felt as if her visit was purely procedural, with no regard for my comfort or privacy. She only wanted to check the cord” [IDI-1; Mother]

However, this experience was not universal. Some parents also highlighted the crucial role of community health worker, who provided emotional and practical support during their time of loss. They recounted illustrating how such support helped them mitigate the difficult times following their loss.

“In that moment, even as my husband was quietly broken and helpless, it was the women around me, my sister-in-law, the ASHA worker, who reacted, who tried to handle things.” [IDI-3; Mother]

Another bereaved father recalled-

“I called her (ASHA) around 1:30 at night when she started having pain, and she was at our home within five minutes. She told me to take her to the hospital and called the ambulance herself. Although her duty hours are normally during the day, she accompanied us all the way to Palwal and returned only around 3:30-4:00 in the morning. The ASHA was with us the whole time. Even after that day, she has been regularly visiting my wife to check on her. ASHA workers are different for each ward. Ours is the best, everyone in our village appreciates her.” [IDI-1; Father]

Although the preceding accounts highlighted both grievances and occasional appreciation for the health system, parents’ experiences of loss extended beyond immediate care or blame. Amidst rituals, grief management, and coping strategies, many continued to seek clarity and understanding about what had happened to their child.

#### 3.2.4 Search for closure

Bereaved parents spoke about different stages of grief, coping practices, and emotional triggers. Across the interviews, many parents referred to a sense of unfinished understanding surrounding the loss. Both mothers and fathers frequently asked, “Why did this happen?” One father reflected on the lack of explanation from healthcare providers,

“The doctor never told me anything. We never got to know the reason why this problem happened. Before it happened, there were no signs, no bleeding, no pain, and no symptoms at all. If there had been even one symptom, we would have known something was wrong. We hoped someone could explain what went wrong.” [IDI-3; Father]

He expressed a strong sense of helplessness, further stating that he had done everything possible to ensure a safe pregnancy.

“She was doing fine, everything was fine. We have a water tank and tap water, a washroom, plenty of fields where she could walk. We also had three ultrasounds, regular check-ups, everything in place. Yet why did this happen? Sir, you are a doctor, can you tell me what could have gone wrong?” [IDI-6; Father]

He described how, despite following all medical advice and precautions, the sudden and unexplained loss left him searching for answers. The absence of a clear reason for the loss added to his distress and prolonged his grief. Similarly, for many parents, the lack of consistent information and the absence of a medical explanation left them feeling confused and abandoned. One father recounted:

“At Hasanpur they said everything was fine, at Hodal also they said the heartbeat was coming. But at Palwal they told us the child had already died. Everywhere they kept saying different things. I don’t know when, why and how did it happen?” [IDI-4; Father]

Silence from medical staff further intensified their distress, as one parent recounted: “The doctors never explained why this happened. They just said these things happen. We kept asking, but there was no answer.”

Even after spending substantial amounts on treatment, parents reported that no effort was made to sit down and provide an explanation:

“We spent more than fifteen thousand in that hospital, but no one even sat with us to explain.” [IDI-4; Mother]

The lack of communication increased their distress and hindered their efforts to understand and cope with the loss. Beyond understanding what went wrong, some parents also expressed that there should be guidance for the health care providers in such circumstances. One father noted:

“There should be someone to guide-What to do, how to take care if such things happen with someone.” [IDI-6; Mother]

## 4 Discussion

This study provides an in-depth account of the lived experiences of PB among parents in Palwal district of Haryana in North India. The study offers a multidimensional understanding of how loss is felt, managed, and negotiated within familial, socio-cultural, and systemic contexts. By employing thematic and Interpretative phenomenological approach, we identified four interconnected themes: (1) parents’ immediate and extended responses to loss, (2) amplifiers of grief at the care provider, familial, and societal levels, (3) coping mechanisms including rituals, memory practices, and blame attribution, and (4) the search for closure. These themes collectively map the multidimensional nature of PB which, along with individual emotions, is also shaped by familial, socio-cultural, and systemic contexts (40). The analysis demonstrates that adaptive grieving in perinatal loss is not about ‘moving on’ but rather about ‘moving forward’ with a continued connection. Our findings also suggests that while Amplifiers of Grief (such as lack of clarity in medical communication) intensify loss-oriented stressors, specific Coping Mechanisms like ‘Ritualized Continuity of Care’ and ‘Memory Making’ serve as vital applications of continuing bonds theory. These bonds facilitate the oscillation between mourning the loss and restoration-oriented adaptation, ultimately helping in reducing the risk of chronic postnatal depressions. These findings contribute to a growing body of qualitative research that foregrounds parental voices in contexts where perinatal loss remains both medically under-acknowledged and socially fraught (41–44). By foregrounding parents’ meaning-making through an interpretative phenomenological lens, the study moves beyond descriptive accounts of grief to illuminate how loss is understood, negotiated, and lived over time.

Parents’ immediate reactions like shock, disbelief, fainting, and inconsolable grief echo findings from other LMIC contexts where perinatal loss is experienced as an abrupt rupture in the moral and emotional order of family life (45, 46). The study also identified gendered differences in how parents experienced perinatal grief and expressed their grief. The findings reveal that both father and mother’s experiences of bereavement are shaped by distinct social expectations and emotional roles. While mothers frequently reported prolonged sadness, headaches, and sleeplessness extending for months, fathers often described burying themselves in work or assuming stoic roles, masking their grief. Such gendered differences in the expression of grief (47–49) resonate with longstanding research on “incongruent grieving” (50), while also reflecting broader cultural expectations around gender in Indian kinship systems that prescribe emotional containment for father and overt suffering for mother. Importantly, some fathers in our study expressed devastation equal to their partners, especially when they had been actively involved during pregnancy. This points to the growing relevance of fatherhood in shaping grief trajectories (51), a phenomenon less explored in South Asian bereavement literature.

Grief was not experienced in isolation; rather, it was exacerbated by systemic, familial, and societal dynamics. Encounters with health systems, such as being left in wards with new mothers (47), the use of dehumanizing terms like “body,” and inadequate communication represented similar experiences documented in other settings (52). The lack of consistent or transparent information about the cause and timing of death was especially distressing, echoing global findings that silence or ambiguity from providers leaves parents suspended in uncertainty (53).

Within families, delayed disclosure of loss to mothers, often justified as “protective,” emerged as a major amplifier of grief. While intended to shield, such secrecy produced feelings of betrayal and exclusion, reinforcing mother’s lack of agency in their own bereavement. Few studies in the South Asian context have documented this specific practice, marking it as a critical contribution of our work. At the societal level, incessant questioning, social avoidance, and stigma compounded maternal suffering, reinforcing findings from studies in sub-Saharan Africa and South Asia where perinatal loss is often viewed as polluting or shameful (54). These dynamics underscore how cultural scripts of secrecy, purity, and social judgments intertwine with bereavement, extending grief far beyond the moment of death.

Parents deployed diverse strategies to manage their grief, reflecting both individual and collective repertoires of coping. Religious rituals and mourning practices such as *puja, hawan and sahada* were central, serving less as pathways to emotional resolution than as socially mandated obligations. In contrast to the similar studies in other regions (55, 56), these rituals provided structure but rarely alleviated pain.

To our knowledge in North Indian setting, we describe a particularly novel finding which we termed as ‘ritualized continuity of care’. It included the embodied practices like offering breast milk at the grave that symbolically extend caregiving beyond death. Such practices resonate with the concept of ‘continuing bonds’ (39) but are uniquely expressed here through culturally sanctioned rituals, highlighting how parents attempt to maintain relationally even after loss. Memory-making emerged as another coping mechanism, with mothers frequently relying on photographs stored on phones or social media. The emergence of *digital remembrance* in this context adds a contemporary dimension to PB studies, where most prior work has focused on tangible mementos (57, 58). In this way, our conceptualization of digital remembrance is similar to Salvini et al. who described bereavement photography and other memory practices as mechanism for enabling continuing bonds, integration of loss, and validation of parenthood (42).

In contrast, withholding memory by deleting photos, discarding belongings, or maintaining silence was an equally striking and underscored the heterogeneity of strategies families use to survive overwhelming grief. This is in accordance with a systematic review that cautioned about the mixed effects of contact/memory making on parents wellbeing and satisfaction (59). Though, the study highlighted that satisfaction with memory-making decisions is often high despite mixed mental-health effects.

Blame attribution, particularly towards healthcare providers, surfaced as both a coping mechanism and a demand for accountability. This is consistent with previous study (45). Fathers especially expressed grievances against doctors and ASHAs for negligence, referral delays, or perfunctory behaviors (60). However, this was not universal as some parents’ also narrated profound appreciation for ASHAs who went beyond their formal duties to accompany families at night or provide follow-up care. These contrasting accounts highlight the ambivalent role of frontline health workers in bereavement both as potential amplifiers of pain and as crucial sources of comfort, depending on the kind of support they provide.

Finally, the findings also highlighted how PB can also influence reproductive decision-making as seen in the case of one father who, framing the survival of one son and one daughter as family “completion,” sought sterilization as a way to secure closure. This decision reflected broader North Indian cultural scripts about the ideal family unit (61), and underscored how PB shapes future reproductive trajectories.

Across narratives, the most persistent theme was the search for closure. The silence and the absence of medical explanations, inconsistent accounts across facilities, and perfunctory reassurances (62)obstructed meaning-making and healing. Even after substantial financial expenditure, parents lamented that no provider took the time to sit with them and explain the cause of their loss. This resonates with global literature documenting the profound consequences of communication failures for bereaved families (51) who described care provider’s behavior to have memorable impacts (63).

The lack of closure not only intensified grief but also left parents anxious about future pregnancies. Several explicitly called for guidance on “what to do, how to take care next time,” highlighting a systemic gap in post-loss counselling and anticipatory care. This gap is especially concerning in the south Asian context, where perinatal mortality remains high (64) and health systems lack structured bereavement care services (48). Along with this, the insights from IDIs with the parents, family and community interviews for social autopsy and positive deviance in the SHRiSTI study highlighted the need to address parental bereavement. Accordingly, we proposed to develop a culturally appropriate bereavement support package with counselling guidelines and an informational guide for healthcare providers to strengthen compassionate care within stillbirth prevention efforts (31, 35).

## 5 Implication of the study

This study highlights the need to strengthen the bereavement care within existing maternal and newborn health services. Parents’ experiences show that small, sensitive actions by health providers can make a profound difference in how families cope with loss. Health facilities can use a simple checklist for managing stillbirths or newborn deaths within 24 hours of birth, focusing on respectful communication and privacy. Key steps include providing a private space for disclosure, using person-first language (avoiding terms such as “body”), giving one consistent explanation of events, allowing time for questions, and offering possible explanation to the cause of their loss before discharge. Wherever possible, bereaved mothers should be kept under care separately from postnatal or breastfeeding areas, even a privacy screen or short-stay room can help reduce distress. Parents should be offered the choice to see and hold their baby or receive simple memory tokens or photographs, if they wish. The existing system of postnatal visits can be strengthened to include emotional and bereavement support, with follow-ups at 24–72 hours and 2–6 weeks to identify distress and provide counselling. Each facility should also maintain a referral map of local mental health and social support resources and use a brief distress screen during follow-up to guide referrals.

## 6 Strengths and limitations of the study

The major strength of the study is the inclusion of narratives of parents, from both mothers and fathers, allowing for a more comprehensive understanding of their experiences surrounding stillbirth and newborn loss. Given the sensitivity of the issue, participant’s comfort was prioritized by using a solace protocol which has been provided in **Supplementary Annexure 3**. Mothers were interviewed by female researchers and fathers by male researchers, which helped build rapport and encouraged open sharing, especially on gendered and emotionally sensitive aspects of grief and coping.

The reflexivity was practiced throughout the research process through regular team debriefs that allowed for collective reflection and critical discussion of emerging insights. These meetings helped in ensuring that interpretations remained grounded in participants’ narratives rather than researcher bias. To strengthen trustworthiness, an audit trail was maintained, documenting key decisions and coding iterations throughout the study. This enhanced the transparency and dependability of the analytic process. Finally, field-note triangulation, combining observational notes, contextual reflections, and interview data, enhanced the credibility and richness of the findings by capturing multiple dimensions of the lived experiences described.

The key limitations of this study include the reliance on ASHAs and facility records for identifying potential participants. This approach may have inadvertently excluded parents who experienced stillbirth or newborn death outside the formal health system, as well as those who were unwilling to revisit their loss due to emotional distress. Such exclusion may have introduced a degree of selection bias and limited the diversity of lived experiences captured in the study. To mitigate this, the study incorporated multiple identification routes and maintained repeated, sensitive contact efforts to enhance inclusion and reduce selection bias. Additionally, repeated and sensitive contact efforts were undertaken to build trust and provide participants with the space and time needed to decide about participation.

## 7 Conclusion

By centering parents’ narratives, this study indicates that PB cannot be reduced to an individual psychological event; rather, it needs to be understood as a relational and culturally embedded process. Bereaved parents often seek explanations for their loss, and the absence of such explanations can lead to a complicated and prolonged grieving period. The study, in the end, highlights the need for sensitizing health care providers about the PB and develop standard bereavement care checklist and protocol to provide emotional support by better communication and ensuring timely follow-up for affected parent and their families.

## Data Availability

The dataset used for this study is derived from a larger dataset collected for multiple research objectives. Only the data relevant to the specific research question addressed in this study has been extracted and analyzed. The datasets generated and analyzed for current manuscript contain sensitive and personal information shared by bereaved parents. To protect participants’ confidentiality and privacy, the full interview transcripts are not publicly available. The thematic framework, representative verbatim excerpts, and the analyzed codebook supporting the study’s findings are provided in the **Supplementary Material** of this article. However, access to the data maybe requested by contacting the Ethics Review Committee (ERC) at the Society for Applied Studies via email at ERC@sas.org.in or by phone at +91-7838350052. The ERC will review data access requests and provide guidance regarding any applicable restriction. More information about the data can be sought from the corresponding author (barsha.pathak@sas.org.in) and/or Sarmila Mazumder (sarmila.mazumder@sas.org.in).
