# Supplementary material for: Lived experiences and coping mechanisms of parents following stillbirth and immediate postnatal death: an interpretative phenomenological study from a North Indian setting
Source: Front Psychiatry. 2026 Mar 2;17:1736722. doi: 10.3389/fpsyt.2026.1736722 (PMC12989504; doi:10.3389/fpsyt.2026.1736722)
Supplement: Supplementary file 1 [file Supplementaryfile1.docx]

Supplementary Material

# Supplementary Data

## Supplementary Annexure 1:

**IN-DEPTH INTERVIEW GUIDE FOR FATHERS EXPERIENCING PERINATAL LOSS**

**Introduction**

Thank you for agreeing to talk with me today. My name is [interviewer name], and I am part of the research team. We are trying to understand the experiences of fathers when a family goes through pregnancy loss. Your thoughts will help us learn about the challenges you faced, the kind of support you needed, and what health workers and families can do better in the future. Everything you share will remain confidential, and there are no right or wrong answers. The conversation will take around one hour. With your permission, I’d like to record so that I can listen carefully rather than take many notes.

Thank you!

1. Could you tell me a little about yourself and your family?
   *(Probe: age, work, family situation, number of children)*
2. How did you feel when you first heard about this pregnancy?
   *(Probe: hopes, expectations, role in the pregnancy, involvement in care decisions)*
3. Did you accompany your wife/partner during check-ups or delivery?
   *(Probe: if yes, in what way; if not, why; interaction with doctors or health workers)*
4. Can you describe what happened when you received the news of the stillbirth/neonatal death?
   *(Probe: Your first response; what went through your mind; did it disturb you; if yes, for how long?)*
5. What was your emotional state in the days immediately after the loss?
   *(Probe: feelings of grief, anger, numbness; moments of disbelief or blame)*
6. Did you feel supported during this period?

(*Probe:* Between you and your spouse, who supported whom most? Who was the main source of support *(spouse, hospital staff, self, parents, no one)?*

1. How did your family, friends, or parents react to your loss?
   *(Did they understand your grief; did you feel acknowledged?)*
2. Were you able to share your feelings openly, or did you feel you had to remain “strong”?
   *(Probe: any pressure to hide emotions; how this affected your grieving)*
3. How did the loss affect your relationship with your wife/partner?
   *(Probe: shared grief, distance, increased closeness, sense of responsibility)*
4. Were you given a chance to see or hold the baby?
   *(Did you participate in rituals, customs, naming, or funeral practices?)*
5. Did anyone for example health workers, relatives, and friends, offer counselling or emotional support?
   *(Probe: What kind; was it helpful; what was missing?)*
6. How did your workplace respond?
   *(Probe: leave, acknowledgement, silence, stigma)*
7. Looking back, what was the most difficult part of this experience for you as a father?
8. What changes would you like to see in hospitals, families, or communities to better support fathers in such situations?
9. Is there anything else that you as a father think people usually don’t talk or ask about?

Thank you for your time and sharing your experience. Your responses will help us in improving support for fathers and guide policies to better care for families facing pregnancy or new born loss in the future.

## Supplementary Annexure 2:

**IN-DEPTH INTERVIEW GUIDE FOR MOTHERS EXPERIENCING PERINATAL LOSS**

**Introduction**

Thank you for taking the time to speak to me today. My name is [interviewer name], and I am one of the SAS team members. Before we begin, can I please confirm that you have received a copy of the study information sheet and consent form?  As a reminder, this project aims to develop a package of interventions that will help to prevent deaths by promoting coverage and quality of the healthcare required during the antenatal period and labor. This form will have questions to understand the bereavement experience and support received by pregnant women with pregnancy loss. During the interview, the questions about the pregnancy experiences, such as what happened during pregnancy, the description of the illness or accident, any complications, the type of care sought and treatment received, and any challenges faced by you in availing the health care services will also be asked. This will help us in strengthening the health systems to prevent such tragedies from happening in the future and highlight the targeted need for bereavement care/counselling after a pregnancy loss.

There are no right or wrong answers. Everything you say will be treated confidentially and will not be shared with anyone outside of our study team.

This interview will take approximately one hour, depending on how much you have to say. Can I please check if you are free now to talk for this amount of time?

I would also like to record our conversation- so that I can capture your responses accurately, and so that I can listen to you rather than take many notes. Can I confirm you are happy for me to start recording?

Thank you

Q. Could you tell me a little about yourself?

[Age, family, work, living situation]

Q. Can you share your journey to this pregnancy?

[Probes: When and how did you learn you were pregnant? How did you get to know about your pregnancy? How was your experience overall? Any specific feeling & emotional change. Perception about her pregnancy: Were there any self-doubts about carrying a recent pregnancy? How did you feel when she shared this news with her husband & family? How do they react? How did you feel during the pregnancy before the loss?]

Q. Please, tell me about your experience with antenatal care. Did you attend antenatal care? How many times did you go? If yes,

[Probes: Where do you go for this (Public/Private, level of facility) and reason? [Timing of the visit, accompanying person, purpose, experience, Gestational month, purpose of visit. If antenatal care was not attended, what reasons? (Accessibility & affordability, multigravida, terrain, poor experience, behavior of healthcare staff); When and where did you go for an ultrasound? What did you learn from ultrasound reports? If you did not have any ultrasound, why?]

Q. Were you aware of any healthcare workers in your area?

[Type of Interactions, support received]

Q. Did you face any complications during your pregnancy?

[Probes: Nature of complications: Early pregnancy: spotting, dizziness, vomiting, diarrhoea, fainting & weakness, APH (fresh blood or old blood); BP, diabetes, constant pain, sign of chlosama; Hypertensive disorders: PIH, Pre-eclampsia, eclampsia, Gestational Diabetes, STI, Hypothyroidism, Hepatitis B, HIV, Malaria]

Q. Have you felt or experienced any moments or at any point of time that something was wrong with your pregnancy? If Yes, when and what?

You can share with me whatever you felt or think that how & why it would have happened? [Probes: Where were you? Who was with you? Spontaneous abortion, stillbirth, neonatal death.]

Q. After the loss, what happened?

[Probes: Were you shown or able to hold the baby/fetus? Was there any naming, blessing, or ritual?]

Q. How have you been coping since the loss?

[Probes: Feelings, thoughts, or changes in daily life. Who have you talked to about it?]

Are there any religious, spiritual, or personal practices you have followed since then?

Q. Did anyone offer you emotional support or counselling?

[Probes: From hospital staff? Family? Friends? Was it helpful?]

Q. How has your body been recovering physically?

[Probes: Did you receive follow-up medical care after the loss?]

Q. How was your health after delivery? Are there anything that you did for betterment for your health?

[Any suggestion/ guidance from the family members, neighbours, peer groups, etc. on what to do after returning home?]

Q. What were you feeling post-delivery after returning home?

[Probes: Did you feel any changes in the behaviour of the family members (husband/ mother-in-laws in particular)]

Q. What kind of support did you receive during the post-delivery period?

[From the family members, friends, doctors, ASHA workers, etc.]

Q. How do people in your community usually talk about such losses?

[Probes: Is it something openly discussed or kept private?]

Q. Are there any customs or rituals in your community for such losses?

[Probes: If yes: Which ones? Did you follow them? Why/why not?

If no: How was the loss acknowledged, if at all?]

Q. Looking back, what was the most difficult part for you?

Q. Is there anything you wish health workers or your family had done differently?

Thank her for sharing her experience.

## Supplementary Annexure 3:

This protocol was developed as part of the study’s ethical safeguards and was approved under the broader ethical clearance of the SHRiSTI project (G Pathak et.al. 2025).

## SOLACE PROTOCOL

The Solace Protocol has been developed to assist and guide the researchers in conducting interviews on sensitive and emotionally charged topics, particularly those involving experiences of stillbirths and neonatal deaths. These interviews were conducted with parents who had recently experienced such losses, with the purpose of understanding not only the clinical but also the social, cultural, and emotional pathways that led to these outcomes.

In the SHRiSTI project, the Social Autopsy interviews were conducted, to understand the broader contextual factors that may have contributed to stillbirths, such as missed or delayed antenatal care (ANC), failure to identify high-risk pregnancies in time, inadequate management of complications, and repeated referrals during the intrapartum period. Understanding these pathways provides critical insight into the barriers that affect maternal and newborn outcomes. In addition, Positive deviance interview were conducted to find the solutions from the community itself by observing the positive behaviours and practices that might have resulted in the positive birth outcome. During these in-depth interviews the process of bereavement interviews were also explored with some extreme cases, to understand how parents process grief, the coping mechanisms they adopt, and whether any bereavement support was provided to them during or after their loss. During all these interviews, researchers found a deeply human perspective on the experience of loss, mourning, and resilience. Many questions were emerged and asked during the interviews. This protocol will highlight those questions and conditions and best possible response which should be addressed by the researchers.

The sensitive topics such as pregnancy loss and neonatal death often evoke profound emotions such as, sadness, anger, fear, and anxiety (H. Westland et al., 2024). The Solace Protocol was therefore developed to ensure that the process of data collection respects the emotional safety, dignity, and autonomy of participants, while allowing them the space to share their experiences if they wish to do so.

Researchers must recognize that interviews on such subjects are not neutral or purely data-driven exercises. They are, instead, deeply human encounters, where empathy, patience, and emotional awareness are as essential as research ethics. The interviewer’s role is to create a compassionate, non-judgmental environment and to respond appropriately if a participant becomes distressed.

**During the Interview: Presence, Language, and Sensitivity**

Interviews should begin with empathy and patience. Participants should be informed that the discussion may be emotional, and that they may pause, skip, or stop at any point.

*“Before we begin, I want you to know that some of the topics we will discuss can be emotional. If at any point you feel uncomfortable, need a break, or would prefer not to answer a question that is completely okay. Please feel free to let me know. Your well-being is very important to us.”*

The interviewer should listen more than they speak, using gentle non-verbal support (a soft tone, slow nods). Phrases like *“I understand”* should be avoided unless based on personal experience; instead, use acknowledgments such as *“Thank you for trusting me with your story.”*

Smith reminds us that *“to interview and then leave someone in emotional distress without adequate support or safeguards is morally wrong”* (Whitney & Evered, 2022). Researchers should therefore debrief with peers or senior colleagues after emotional interviews to process their own reactions and prevent burnout.

**Expected Questions & Scenarios during the Data Collection**

| **Questions & Scenarios during the Data Collection** | **Suggestions & Statement for the Interviewer** |
| --- | --- |
| **Initiate the Interview** with empathy, patience, and flexibility. | *Before we begin, I want you to know that some of the topics we will discuss can be emotional. If at any point you feel uncomfortable, need a break, or would prefer not to answer a question, that is completely okay. Please feel free to let me know. Your well-being is very important to us.* |
| **Women are concerned about whether they will experience a similar pregnancy loss again in the future** | *“Yes, we understand your concern, and this is a valid question. I feel your obstetrician is the best person to ask this question since they understand your case better and they will help you better prepare for your next pregnancy.”*  “हाँ, हम समझते हैं कि आपके जो सवाल या चिंताएँ हैं, वो बिल्कुल सही हैं। लेकिन इस बारे में जिस डॉक्टर को आप दिखा रही हैं (प्रसूति विशेषज्ञ / Obstetrician), उनसे पूछना बेहतर रहेगा, क्योंकि वे आपके केस को बेहतर तरीके से समझते हैं और आने वाली प्रेग्नेंसी में ऐसी दिक्कत न हो, उसके लिए आपको अच्छी तरह तैयार करेंगे।” |
| **Family members ask, “Now the baby is no more, how can this interview help us? we had visited the hospital a number of times, and nobody took care of us.”** | *“We understand that you are upset by your experience at the facility, but what information you give us today will help the government in preventing such deaths in the future by making improvements in the services provided during pregnancy and at the time of delivery”*  “हम समझते हैं कि आपको अस्पताल में कुछ दिक्कतें आई थीं, लेकिन आज जो जानकारी आप हमें देंगी, उससे सरकार को यह समझने में मदद मिलेगी कि गर्भावस्था और प्रसव के दौरान देखभाल में किन चीज़ों में सुधार की ज़रूरत है।” |
| **First-time pregnant women are generally more hesitant to talk about the death of the baby, compared to women who have had babies in the past. Other family members responded in her place. How to encourage and convince the family members so that we can get the woman herself to talk about her experience** | *Instructions for Researchers – First listen to everything the family members have to say respectfully. Then request them to let us talk to the woman (if possible, alone) because certain emotions and experiences that the woman has gone through, no one can explain these things better than her, and we would prefer to hear directly from her.*  **Researchers के लिए निर्देश –** “पहले परिवार के सदस्यों की सारी बातें सम्मानपूर्वक सुनें। फिर उनसे निवेदन करें कि वे हमें महिला से बात करने की अनुमति दें (यदि संभव हो तो अकेले में), क्योंकि ये भावनाएँ और अनुभव जिनसे महिला गुज़री है, उन्हें उससे बेहतर कोई और नहीं समझ सकता। हम सीधे उसी से सुनना पसंद करेंगे।”  ***Possible follow-up questions: What kind of emotions or experiences do you want to know about?***  *“Because we want to understand her feeling of loss and ensure her well-being”*  **संभावित फॉलो-अप सवाल:** “आप ऐसी किन तरह की भावनाओं या अनुभवों के बारे में जानना चाहते हैं?”  **उत्तर/स्पष्टीकरण के रूप में कहा जा सकता है,** “क्योंकि बच्चे को खोने की जो भावना महिला ने महसूस की है, हम उसकी स्थिति को बेहतर समझना चाहते हैं और यह सुनिश्चित करना चाहते हैं कि उसकी पूरी सहमति और भलाई बनी रहे।” |
| **Sometimes family members (husband, father-in-law, and mother-in-law) say, “We can give you any information you need, the baby has passed away, we don’t want you to ask her pressing questions”. How to handle this situation?** | *In this case, we have to read the room, and we can approach it two ways –*  *If the family prefers, it would be better if the visit takes place in a few days, as the woman will be in a better condition to answer questions.*  *We can ask the family member to also be present during the interview for their satisfaction.*  ऐसी स्थिति में, उस समय के माहौल के अनुसार जवाब दें। यदि परिवार चाहता है कि यह विज़िट कुछ दिनों बाद हो, ताकि महिला सवालों के जवाब देने के लिए बेहतर स्थिति में हो, तो हम उनकी बात मान सकते हैं।  हम परिवार के सदस्यों को उनकी संतुष्टि के लिए यह भी कह सकते हैं कि वे इंटरव्यू के दौरान उपस्थित रह सकते हैं।” |
| **In some cases, the ladies say that when they got admitted for delivery at that time FHR was checked and the nurse told them the heartbeat is present. But the baby was born stillborn.** | *Even at the time of delivery there can be complications that can result in the baby being born stillbirth.*  “कई मामलों में, जिनमें गर्भावस्था के दौरान कोई दिक्कत नहीं होती, उनमें भी डिलीवरी के समय परेशानियाँ उत्पन्न हो सकती हैं।” |
| **In some cases, women show post-pregnancy symptoms, and they turn to the FA for advice** | *Yes, we understand your concern, and this is a valid question. In case symptoms are observed post-delivery you should consult your obstetrician since they know your case better and can help manage symptoms or provide treatment if required*  हाँ, हम आपकी चिंता को समझते हैं, और आपका सवाल सही है। यदि प्रसव के बाद कोई लक्षण दिखाई दें, तो आपको अपने प्रसूति रोग विशेषज्ञ से परामर्श करना चाहिए, क्योंकि वे आपकी प्रेग्नेंसी को बेहतर जानते हैं और लक्षणों को रोकने में मदद कर सकते हैं। यदि आवश्यक हो, तो वे उपचार भी प्रदान कर सकते हैं।” |
| **During the Interview:**  ***Key Signs of Emotional Distress***   1. Crying 2. Becoming very quiet or withdrawn 3. Shaking, breathing heavily, or visible distress 4. Expressing feelings of guilt, anger, or hopelessness | *Recognize that crying or emotional moments are normal and do not necessarily mean the interview must stop.* |
| **If the Participant Becomes Emotional** | Allow the participant to set the pace and offer breaks if needed.  **Pause**: Stop asking questions.  **Acknowledge their emotion**: Gently recognize and validate their feelings.  **Offer comfort**: Allow silence; offer tissues if available.  **Assurance of confidentiality:** before resuming the interview.  **Give control back**: Ask if they would like to pause, continue, or stop.  **Respect their decision**: Never pressure them to continue. |
| Always prioritize the participant's well-being over completing the interview, but in case the participants don’t want to or are not in a state of sharing anything, either the interview should be stopped immediately, or the interviewer should show some empathy towards the participants by using the script provided for the interviewer | **Interviewer Script**  “मुझे पता है कि इस बारे में बात करना बहुत मुश्किल है। कृपया अपना समय लें।”  "*I know this is very difficult to talk about. Please take your time."*  *"It's okay to feel emotional. Would you like to take a break, have a moment, or continue when you’re ready?"*  “भावनात्मक होना बिल्कुल ठीक है। क्या आप ब्रेक लेना चाहेंगी, थोड़ी देर रुकना चाहेंगी, या जब आप तैयार हों तब जारी रखना चाहेंगी?”  *"Thank you for sharing such a deeply personal experience. We can pause here if you would like."*  “इतना व्यक्तिगत अनुभव साझा करने के लिए धन्यवाद। यदि आप चाहें तो हम यहां रुक सकते हैं।” |
| **If the Participant Wants to Stop** | *Thank them for their courage and time.*  “आपके साहस और समय के लिए धन्यवाद।”  *Gently ask if they would like to share anything.*  “क्या आप कुछ और साझा/बताना करना चाहेंगी?”  *Close the interview respectfully.* “धन्यवाद। हम अब इंटरव्यू को सम्मानपूर्वक समाप्त करते हैं।” |
| **Closing Statement** | *"Thank you again for speaking with me today. I truly appreciate you sharing your experiences. Please remember to take care of yourself.”*  “आज मुझसे बात करने के लिए आपका फिर से धन्यवाद। मैं वास्तव में आपके अनुभव साझा करने की सराहना करती/करता हूँ। कृपया अपना ध्यान रखना न भूलें।” |

**Recognizing and Responding to Emotional Distress**

Common signs of distress include crying, sudden silence, heavy breathing, shaking, or expressions of guilt, anger, or hopelessness. These are natural reactions and do not automatically mean the interview must stop.

When distress appears:

- Pause questioning and allow silence.
- Offer water or tissues.
- Acknowledge the emotion gently.
- Give the participant control over how to proceed.

*“I know this is very difficult to talk about. Please take your time.”*
*“It’s okay to feel emotional. Would you like to take a break, have a moment, or continue when you’re ready?”*
*“Thank you for sharing such a deeply personal experience. We can pause here if you would like.”*

If the participant chooses to stop:

- Thank them for their courage and time.
- Ask softly if they would like to share anything else.
- End the conversation respectfully.

*“Thank you again for speaking with me today. I truly appreciate you sharing your experiences. Please remember to take care of yourself.”*

**Quick Responses & Action**

| **Phases** | **Actions** | **Key Phrases** |
| --- | --- | --- |
| Recognize | Notice signs of distress | “I can understand this is difficult for you.” |
| Pause | Stop questioning | Silent support |
| Acknowledge | Validate their emotion | “It’s okay to feel this way.” |
| Offer Comfort | Allow silence, offer tissues | “Take your time.” |
| Reassure | Remind of confidentiality | “You’re in control of what you share.” |
| Give Control | Offer choice | “Would you like to pause or continue?” |
| Respect | Follow their lead | “Thank you for sharing that.” |

**Referral and Support Mechanisms**

Researchers should be prepared to identify participants showing ongoing distress, symptoms of depression, or those reporting domestic violence. In such cases, a **referral mechanism** must be activated.

- Provide information on local mental health or women’s helpline services (e.g., Tele MANAS: 14416 & 1800-891-4416, *Women’s Helpline 181*).
- Explain that support is available and confidential.
- Do not pressure or insist that offering help is enough.
- In cases of immediate risk (self-harm, violence), notify the study supervisor the same day.

**After the Interview**

Researchers must also care for themselves. Emotional interviews can be draining. Field investigators are encouraged to debrief with a senior team member and take time to process their own reactions. Conducting multiple grief-related interviews in one day should be avoided.

**Conclusion**

The *Solace Protocol* is not just a set of interviewing rules it is a commitment to empathy. Sensitive research requires compassion as much as methodological rigor. Each encounter is an ethical space where respect, patience, and human connection matter as much as data. By following these principles, researchers can ensure that no participant and no researcher leaves an interview emotionally unsupported.

# Supplementary Table

# 2.1 Supplementary Table 1: THEME WISE NARRATIVES OF BEREAVED PARENTS

| **Theme** | **Narratives of Bereaved Parents** |
| --- | --- |
| **Response** | “Everyone is distressed. But mostly the mother suffers more. You also know men neither cry openly nor show it. That’s how it is. Most mother express grief. Men just sit quietly, looking sad.” [IDI-1; Father]  “Since I am the elder in the house and there is no one else above me, I had to understand it.” [IDI-2; Father]  “Look, anyone feels sorrow when a child dies. After living together, eating together, staying together throughout the pregnancy, of course, both mother and father feel the loss. The whole family feels it.” [IDI-1; Mother]  “There is this PHC near our village. I used to go with her every month for check-ups. We also gave reports to the Anganwadi worker. It was never that we delayed it by a month or so, we went every month without fail. Because we didn’t even have one percent of doubt, we had gone so happily, like it was just another normal check-up. And then suddenly they told us there was this problem…. we couldn’t believe it. But when the second report came back around 10:30-11 am, the doctor said the baby was no more. You will have to get an abortion as soon as possible. I could not believe it. I felt like the ground fell out from beneath my feet. How could it happen? I was shattered. We both came out and started crying.” [IDI-3; Father]  “I keep thinking that it was such a good boy. If it had been a girl, she might have survived.” [IDI-3; Mother] |
|  |  |
| **Amplifiers of Grief** | “I just couldn't stay there... seeing them made me want to cry a lot... because I didn't have my child... I didn't tell anyone anything... No, I didn't tell anyone; I only kept telling my husband and my sister-in-law again and again that take me away from here, I don't feel comfortable here, everyone here has children and I am like this here. Then my husband was saying we will leave for home only when the doctor gives leave.” [IDI-5; Mother]  “After the delivery, all the mothers in the hospital were breastfeeding their babies. Seeing them gave me so much pain, I kept thinking, I would have been feeding my baby like that too. I was a mother who had just lost her child, surrounded by others holding theirs, breastfeeding them.” [IDI-8; Mother]  “We spent more than fifteen thousand in that hospital, but no one even sat with us to explain. This made me so angry and helpless at the same time.” [IDI- 2; Father]  “The nurse wrapped the baby in a swapy (baby sheet) and kept it on a tray. Then she told me that she (mother) need to be kept under observation for 12 more hour. Please take the “body” from here. It was around 12 in the night. How could we take home right now? Unfortunately, that day, there was too much rain and thunder that night. I said where will we go right now? It is too heavily raining outside, can we take it when the rain stops? But the nurse was “not good”. She looked at the tray where the baby was kept and said “body cannot be kept here whole night.” I tried to explain her that our house was 10 km away, we would take it as soon as the rain stops in the early morning but she wasn’t ready to listen.” [IDI-3; Father]  “I wasn’t there at the time. If I had been there, I would not have taken the baby to her (the nurse). I would have gone straight to the government hospital.” [IDI-1; Father]  “Two days after coming from the hospital, I overheard my sister in law and mother in law talking with each other. That’s when I learnt about it. Since then, I kept crying I kept saying to myself that this was wrong, that they should have told me earlier. At that moment I felt so hurt; I kept thinking if they had told me sooner, maybe it wouldn’t have been so painful, maybe I could have handled it. They had hidden it, perhaps to spare me, but instead it felt like a betrayal. I didn’t even see or hold the baby once, not even once. Sometimes I think, if I had at least held it, I might have known what it would have been like; perhaps I would have cried differently, perhaps the images in my head wouldn’t keep coming back. Now I keep going over that day in my mind, again and again; some days it overwhelms me.” [IDI-2; Mother]  “I used to feel restless, as if I should go far away from here. Everyone was around me-my sister-in-law was there with me all the time, my husband’s aunt and my mother-in-law also stayed in my room. But still my mind didn’t settle with their conversations. They kept me engaged in their talk, yet I still felt like staying apart from them. They would often explain to me, saying, ‘Babita (changed name), don’t think so much. You will regret it later. Your eyes will hurt, your head will ache-your head is still fragile, your eyes are delicate. Don’t think so much.’" [IDI-10; Mother]  “I am unable to digest this. It just doesn't go from my mind. Our burial ground is just on the way to my work, dragging my attention each time I pass through that route. In the initial few days, I unconsciously went to the grave just to check if the grave was safe. I was scared that the stray animals might dig it up or remove the soil from it. And in general I used to walk through that way for many days whenever this thought came to my mind.” [IDI-1; Father] |
| **Coping mechanisms** | “For a small child, the rituals usually last only 3-4 days. For adults, it goes on longer upto 13 days. We had to perform the rites. We performed a ‘Hawan’ and sprinkled holy water from Ganga in the house, that’s all. All of this was done on fifth day. Until then, we were at home, didn’t go anywhere. During that time, food wasn’t cooked at home for a day or two, maybe 2 or 3 days. The ‘Puja’ was probably done on the 5th day. After that, from the next day, we resumed our usual work.” [IDI-6; Mother]  “It didn’t feel like anything at all… it just felt like our daughter suddenly disappeared. People do puja and rituals for peace of mind, but it’s not like it helps you forget. That peace of mind doesn’t really come from anyone. When someone passes, whether a child or an adult, you still have to follow the rituals that are prescribed. Otherwise, people start talking: this wasn’t done, that wasn’t done. But the truth is, the pain doesn’t go away. Yes, maybe the grief for an adult is felt more deeply, and for a child, maybe a little less, but the sorrow remains either way.” [IDI-3; Mother]  “After the baby passed away, we performed the ‘hawan’. My breast milk was then taken to the cremation ground where the child was buried. The priest had also advised us to do this during the ritual, reminding us that the soul of a baby should not suffer from hunger. So both at the time of burial, and again on the second and third days, my husband went to the cremation ground and placed the milk at the grave.” [IDI-6; Mother]  “I have never gone there, not even once. In our culture, women are not allowed to go. It gave a little peace of mind, to feel that even in death, we were still feeding our child. My husband, though silent, supported every ritual, making sure it was done properly. For him too, it was a way of showing care, even though the baby was no longer with us.” [IDI-8; Mother]  “In our deen (faith), it is believed that whatever happens is for the best. According to our beliefs, a child below the age of eight is considered to go straight to ‘jannat’ (heaven), he is not held accountable like adults, who face either heaven or hell. So we are taught to accept it with patience. But still, it's true that everyone longs to hold the child just once, to feel that embrace.” [IDI-8; Father]  “I just thought I already had one son and one daughter, so I got the operation done. I said to my wife, we already have two children… what else is there to do now? Let’s get the operation done. But the doctor refused to do it now, otherwise I would have gotten it done. Now there are two children, and we have to think only about them.” [IDI-1; Father]  *“My bond with her is stronger now, more than before. Because when you see a person go through such a situation, like I saw her in the hospital, then only you truly understand them.”* [IDI-3; Father] |
| **Memory Making** | “What if she is not here? At least I have her memories with me. Whenever I feel low or miss her, I see her picture which I had posted on Instagram after she was born.” [IDI-13; Mother]  “We performed a ‘hawan’ at home after the baby passed away. Since I was still in my postpartum period and considered ‘unclean’, the photo of the baby was removed from where it was kept and placed in another room. I asked for the photo to be left with me. My husband understands this attachment. Even though the elders follow traditions of purity and rituals, he quietly supported my wish to keep the baby’s memory alive in our home.” [IDI-7; Mother]  “All her clothes, medicines, tablets, anklets, whatever was there, we removed it all from our house. After performing the last rites, when I returned home, I tore up and threw away all the papers, reports, ultrasounds, everything. The clothes and the bed sheet were given to the bhangi (a term used for the sweeper). Whatever the child had used, we put it all out. This is what happens with the material memory of everyone in our culture, whether it is a child or an adult.” [IDI-1, Father]  “I deleted the photos that were on my phone, but the ones that had been put on Instagram at the time, those won’t get deleted so easily. Then, the photos I had taken to send to the nurse were also there. I removed all of them. I didn’t like seeing her crying all the time by seeing those pictures in the phone.” [IDI- 1, Father]  “We had been coming for regular check-ups so many times, but she never checked the heartbeat. It was only when the child died that they checked the heartbeat. Our child could have been saved if they had regularly checked the heartbeat during the previous visits.” [IDI-6; Mother]  “She came only after getting instructions from above. I was still in the bathroom when she arrived, and she didn’t even step inside the house. She simply glanced at the child, quickly jotted something in her register, and left. While I was bathing, she even forced the gate open and didn’t allow me to finish properly. It felt as if her visit was purely procedural, with no regard for my comfort or privacy. She only wanted to check the cord” [IDI-1; Mother]  “In that moment, even as my husband was quietly broken and helpless, it was the women around me, my sister-in-law, the ASHA worker, who reacted, who tried to handle things.” [IDI-3; Mother]  “I called her (ASHA) around 1:30 at night when she started having pain, and she was at our home within five minutes. She told me to take her to the hospital and called the ambulance herself. Although her duty hours are normally during the day, she accompanied us all the way to Palwal and returned only around 3:30-4:00 in the morning. The ASHA was with us the whole time. Even after that day, she has been regularly visiting my wife to check on her. ASHA workers are different for each ward. Ours is the best, everyone in our village appreciates her.” [IDI-1; Father] |
| Search for Closure | “The doctor never told me anything. We never got to know the reason why this problem happened. Before it happened, there were no signs, no bleeding, no pain, and no symptoms at all. If there had been even one symptom, we would have known something was wrong. We hoped someone could explain what went wrong.” [IDI-3; Father]  “She was doing fine, everything was fine. We have a water tank and tap water, a washroom, plenty of fields where she could walk. We also had three ultrasounds, regular check-ups, everything in place. Yet why did this happen? Sir, you are a doctor, can you tell me what could have gone wrong?” [IDI-6; Father]  “At Hasanpur they said everything was fine, at Hodal also they said the heartbeat was coming. But at Palwal they told us the child had already died. Everywhere they kept saying different things. I don’t know when, why and how did it happen?” [IDI-4; Father]  “We spent more than fifteen thousand in that hospital, but no one even sat with us to explain.” [IDI-4; Mother]  “There should be someone to guide-What to do, how to take care if such things happen with someone.” [IDI-6; Mother] |

**2.2 Supplementary Table 2: COREQ (COnsolidated criteria for REporting Qualitative research) Checklist**

| **Topic** | **Item No.** | **Guide Questions/Description** | **Reported on**  **Page No.** |
| --- | --- | --- | --- |
| Domain 1: Research team  and reflexivity | | | |
| Personal characteristics | | | |
| Interviewer/facilitator | 1 | Which author/s conducted the interview or focus group? | Page no. 9 &36 |
| Credentials | 2 | What were the researcher’s credentials? E.g. PhD, MD | Page no. 9 & 10 |
| Occupation | 3 | What was their occupation at the time of the study? | Page no. 9 & 10 |
| Gender | 4 | Was the researcher male or female? | Page no. 9, 12 & 36 |
| Experience and training | 5 | What experience or training did the researcher have? | Page no. 9 |
| Relationship with participants | | | |
| Relationship established | 6 | Was a relationship established prior to study commencement? | No prior relationship was established between the researcher and participants before the study commenced |
| Participant knowledge of  the interviewer | 7 | What did the participants know about the researcher? e.g. personal goals, reasons for doing the research | Page no. 9, 10 & 13 |
| Interviewer characteristics | 8 | What characteristics were reported about the inter viewer/facilitator?  e.g. Bias, assumptions, reasons and interests in the research topic | Page no. 11& 12 |
| Domain 2: Study design | | | |
| Theoretical framework | | | |
| Methodological orientation and Theory | 9 | What methodological orientation was stated to underpin the study? e.g. grounded theory, discourse analysis, ethnography, phenomenology,  content analysis | Page no.7 &15 [Interpretative Phenomenological Analysis (IPA)] |
| Participant selection | | | |
| Sampling | 10 | How were participants selected? e.g. purposive, convenience,  consecutive, snowball | Page no. 8 [Purposive Sampling] |
| Method of approach | 11 | How were participants approached? e.g. face-to-face, telephone, mail,  Email | Page no. 9 & 10 [Telephonic & face-to-face] |
| Sample size | 12 | How many participants were in the study? | Page no. [Total in-depth interviews =30 (15 Mothers & 15 Fathers)] |
| Non-participation | 13 | How many people refused to participate or dropped out? Reasons? | Page no. 8 [researchers have mentioned the inclusion & exclusion criteria] |
| Setting | | | |
| Setting of data collection | 14 | Where was the data collected? e.g. home, clinic, workplace | Page no. 10 [Home, Workplace or any other place of participant’s choice] |
| Presence of non-  participants | 15 | Was anyone else present besides the participants and researchers? | Page no. 13 |
| Description of sample | 16 | What are the important characteristics of the sample? e.g. demographic  data, date | Page no. 15 [Socio-demographic characteristics provided in text as well as in Table 1] |
| Data collection | | | |
| Interview guide | 17 | Were questions, prompts, guides provided by the authors? Was it pilot  tested? | Page no. 11 |
| Repeat interviews | 18 | Were repeat interviews carried out? If yes, how many? | No |
| Audio/visual recording | 19 | Did the research use audio or visual recording to collect the data? | Page no. 10, 13 & 46 [Audio recording of In-depth interviews] |
| Field notes | 20 | Were field notes made during and/or after the interview or focus group? | Page no. 9 |
| Duration | 21 | What was the duration of the interviews or focus group? | Page no. 10 |
| Data saturation | 22 | Was data saturation discussed? | Page no. 10 [Idiographic completeness was achieved] |
| Transcripts returned | 23 | Were transcripts returned to participants for comment and/or correction? | No |
| **Domain 3: analysis and**  **Findings** |  |  |  |
| *Data analysis* |  |  |  |
| Number of data coders | 24 | How many data coders coded the data? | Page no. 7, 14,15 & 38 [Yes, 2 researchers were involved during the coding of transcripts] |
| Description of the coding  tree | 25 | Did the authors describe the coding tree? | Page. 16 [Yes, In Table 2 & Table 3] |
| Derivation of themes | 26 | Were themes identified in advance or derived from the data? | Page no. 16 [Both, Deductive thematic analysis & IPA] |
| Software | 27 | What software, if applicable, was used to manage the data? | Page no. 13 [Yes, NVivo Software version 15] |
| Participant checking | 28 | Did participants provide feedback on the findings? | No |
| *Reporting* |  |  |  |
| Quotations presented | 29 | Were participant quotations presented to illustrate the themes/findings?  Was each quotation identified? e.g. participant number | Yes, Page no. 16 to 31 |
| Data and findings consistent | 30 | Was there consistency between the data presented and the findings? | Yes, Page no. 2, 16, 31 |
| Clarity of major themes | 31 | Were major themes clearly presented in the findings? | Yes, Page no. 16 to 31 |
| Clarity of minor themes | 32 | Is there a description of diverse cases or a discussion of minor themes? | Yes, Page no. 7 & 13 |
